# Modifiable factors associated with weight regain after bariatric surgery: a scoping review

**DOI:** 10.12688/f1000research.18787.2

**Published:** 2020-09-03

**Authors:** Lisa Kaouk, Amy T. Hsu, Peter Tanuseputro, Mahsa Jessri

**Affiliations:** 1 McGill University Health Centre, 1001 Decarie Blvd, Montreal, QC H4A 3J1, Canada; 2 Ottawa Hospital Research Institute, 1053 Carling Ave, Ottawa, ON K1Y 4E9, Canada; 3 Bruyère Research Institute, Ottawa, Ontario, Canada; 4 Department of Medicine, University of Ottawa, Ottawa, Ontario, Canada; 5 School of Epidemiology and Public Health, University of Ottawa, Ottawa, Canada

**Keywords:** Bariatric surgery, weight loss surgery, weight regain, weight recidivism, modifiable behaviors

## Abstract

**Background:** Although bariatric surgery is the most effective treatment for severe obesity, weight regain may still occur. While non-modifiable factors associated with weight regain have been explored, modifiable factors responsible for weight regain are understudied. This scoping review aimed to identify modifiable behaviors associated with weight regain after bariatric surgery.

**Methods:** A systematic search was conducted in Medline, Google Scholar, Cochrane, National Collaborating Centre for Methods and Tools (NCCMT) and Practice-based Evidence in Nutrition (PEN) which included articles published between January 1990 and February 2 2017, for studies examining “weight regain” after bariatric surgery. A total of 293 citations were retrieved. Eligible articles must have examined modifiable factors and addressed weight regain, or a long-term post-operative phase in which weight regain may occur. After removing duplicates, 22 studies were included for thematic analysis.

**Results: **Key modifiable factors associated with weight regain were identified and categorized under the following themes: poor dietary adherence (e.g. excessive calorie, carbohydrate, and alcohol intake), maladaptive eating behaviors (e.g. grazing, binging), lack of on-going follow-up with the bariatric team and insufficient physical activity.

**Conclusions: ** Health professionals and self-monitoring tools for patients who have undergone bariatric surgery may benefit from these findings to direct their education and interventions to target behavior change.

## Abbreviations

BMI: body mass index, RYGB: Roux-en-Y gastric bypass, EWL: excess weight loss, BED: binge eating disorder, LAGB: laparoscopic adjustable gastric band, AGB: adjustable gastric band, VBG: vertical banded gastroplasty, QOL: quality of life, CBT: cognitive behavioral therapy, DS: duodenal switch, ASMBS: American Society for Bariatric and Metabolic Surgery.

## Introduction

Severe obesity, measured by a body mass index (BMI) ≥35 kg/m ^2^, is a complex, multifactorial disease that has been shown to significantly increase the risks of morbidity (e.g. cardiovascular diseases, type 2 diabetes, cancers) and mortality ^1^. Before discussing bariatric surgery, it would be important to first address obesity as a complex chronic disease which requires several interventions over the course one a person’s lifetime in an effort to treat it. The Obesity Medicine Association (OMA) defines obesity as “a chronic, relapsing, multifactorial, neurobehavioral disease, wherein an increase in body fat promotes adipose tissue dysfunction and abnormal fat mass physical forces, resulting in adverse metabolic, biomechanical, and psychosocial health consequences”. As such, the treatment of obesity requires a multifaceted approach customized to each individual’s needs including treatment options such as lifestyle modifications, pharmacotherapy, and surgery, are valuable treatments. Bariatric surgery has been established as a proven treatment option for severe obesity and shown to be successful in achieving varying degrees of weight loss, health gain (including reduced morbidity and mortality), improved mental health, and quality of life ^2^. However, the sustained health improvements following bariatric surgery are dependent on the individual’s adherence to long-term changes in lifestyle habits ^3^. As a result, despite its effectiveness, weight regain after bariatric surgery is still possible. There are two distinct patient populations observed with people who had bariatric surgery with suboptimal or poor outcomes. There are those who do not lose the expected or anticipated average percentage of weight following surgery, while there are others who lose a successful amount of weight but who regain some, or most, of the weight they had initially lost via bariatric surgery. This scoping review observes the latter group of people.

Studies have estimated an average of 56% of patients regain weight within ten years of their surgery ^3^, and about one in four fail to achieve the average expected weight loss and begin to regain weight from their lowest post-operative weight, following the first post-operative year. On average, individuals will achieve 20 – 30% of total weight loss at one to two years post-operative ^4^, and can regain an average of 7% of their total body weight from their lowest post-operative weight over the course of 10 years ^2, 5, 6^. Among patients who have had Roux-en-Y gastric bypass (RYGB), about 15% regain between 2 – 5% of weight from their lowest reported post-operative weight (nadir weight) within two years of surgery, which has been reported to increase to 70% of patients between two and five years, and 85% at over five years post-surgery ^3^. The high prevalence of weight regain after bariatric surgery has resulted in a significant increase in revisional bariatric surgery ^6^, which bears an increase in surgical risk and adverse outcomes to the patient ^7^.

Despite the prominence of weight regain following bariatric surgery, the underlying reasons for weight regain are not well-understood, but have been attributed to a number of surgical, biological and behavioral factors ^8^. Although non-modifiable factors (e.g. hormonal, metabolic, surgery-related) have been identified in the literature ^8^, less attention has been given to the modifiable behaviors and practices that could be implemented by patients and health care professionals. The primary objective of this scoping review was to identify the modifiable factors associated with weight regain following bariatric surgery. A secondary objective of this scoping review was to identify gaps and limitations of existing studies and evidence, which may provide guidance on areas of future research. We followed guidelines of Colquhoun *et al.* ^9^, which is based on the Arksey and O’Malley framework ^10^, for conducting and reporting of scoping reviews.

## Methods

### Search strategy

A systematic search of the literature was conducted in Medline, Google Scholar, Cochrane, National Collaborating Centre for Methods and Tools (NCCMT), and Practice-based Evidence in Nutrition (PEN). The most recent search was conducted on February 2, 2017. We included studies published in English between January 1990 and January 2017, using Boolean search terms—such as, “weight loss maintenance” OR “weight regain” AND “behavior” AND “English” AND “adult” (see *Extended data*) - that were identified by the research team ^11^. An example of the search strategy used in Medline includes the following search terms: ("weight loss maintenance"[All Fields] OR "weight regain"[All Fields]) AND "behavior"[All Fields] AND (("1990/01/01"[PDAT] : "2017/02/02"[PDAT]) AND "humans"[MeSH Terms] AND English[lang] AND medline[sb] AND "adult"[MeSH Terms]). Manual searches of cited references were also conducted to identify additional articles, as described in the eligibility criteria explained below. In total, 276 studies were identified using this search strategy once duplicates were removed.

### Inclusion and exclusion criteria

**Figure 1.**
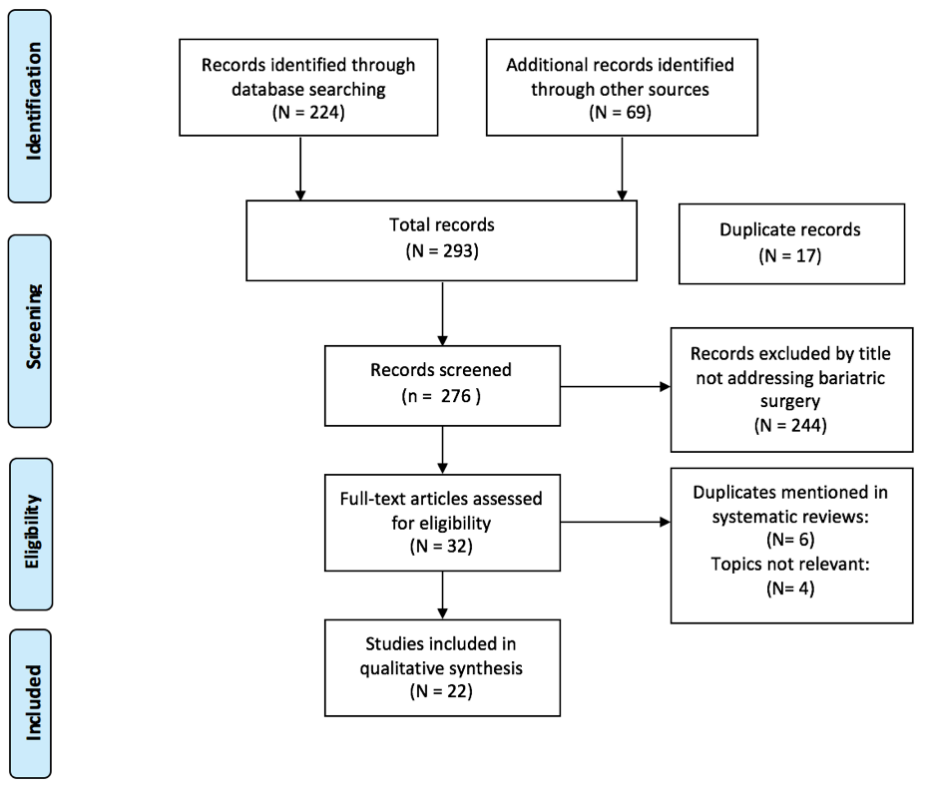
PRISMA flowchart. This PRISMA flowchart depicts the number of records identified through database searching and other sources, and the final number of articles – 22 articles – included in this scoping review.

Our inclusion criteria were any published studies, reviews, practice guidelines and expert opinions involving adults (18+ years) in English between January 1, 1990 and January 31, 2017. All studies and reviews published after this date were excluded. To further narrow the number of papers for full-text review, only titles and/or abstracts containing the terms “bariatric surgery” or “weight loss surgery”, or an association of these terms, were included (n=32). All bariatric surgery types were considered, even those no longer being commonly performed (e.g. vertical banded gastroplasty, VBG). Given that the research community has not yet reached consensus on how weight regain is defined or compared, all studies addressing weight regain either as a percentage of regain or in weight gained were included regardless of the extent of regain. We retained studies that examined modifiable factors (such as diet, behavior/psychology, support, and physical activity) either exclusively or in conjunction with other influences. Studies that focused solely on non-modifiable factors including, but not limited to, gastric pouch size, age, sex, and pre-operative body mass index (BMI), were excluded. The included studies must have identified or referenced weight regain, or explored a period of possible weight regain or weight maintenance; however, no limit in time-frame was applied. Studies that only addressed insufficient weight loss without addressing weight regain were excluded. A total of 32 full-text reviews were performed and 22 articles were included for thematic analysis, after being assessed for relevancy and removing articles mentioned in the systematic reviews that were included (see Figure 1).

Data was charted into an excel spreadsheet. This data was gathered, charted, and inputted independently, and reviewed by two other authors (AH, MJ). All authors discussed the themes that emerged in order to define the variables. Emerging themes that were factors in weight regain were documented and grouped into variables that summarized the themes, namely into behavioral, environmental, support, and exercise. All articles were assigned and coded to one, or more, of the stated variables.

## Results

After removing duplicates (n = 17), 276 articles were screened by their titles, followed by their abstracts. After excluding the articles that did not meet our inclusion criteria, 32 articles remained. Articles were further excluded if they were not relevant to weight regain post-bariatric surgery, involved clinical practice guidelines that did not explicitly examine measurable outcomes, and if they were already mentioned in the included systematic reviews. A total of 22 full-text articles were included in the final synthesis of this scoping review. We extracted information on each study’s authors, location, design, scope of the evaluation (i.e. type of surgery, characteristics of the patient population, modifiable behaviors examined), outcome metrics and key findings (see Table 1).

**Table 1.** Summary of results.

Author Year Country	Bariatric procedure & modifiable behavior	Study design & population studied	Intervention/Group comparisons	Main results
Alvarez 2016 Chile	Sleeve gastrectomy D, B	Case control of 40 participants, 24 -55 months post-op, 80% female, avg 43 years old; pre-op BMI 35 kg/m^2. Self-reported food intake and standardized psychological questionnaires, and some guided.	2 groups: >50th %ile weight regain and <50th %ile weight regain, by 2 years post-op.	Group with higher vs lower weight regain: + 11.05 kg vs +3.55 kg. (p = .00). Gastric volume (p = .023), higher fat (p = .04) and energy intake (p = .09), and post-operative higher anxiety (p = .01) are associated with weight regain. Alcohol consumption (p = .81), fat as % total calories (p = .28), BED, depression, and exercise (p = .49) were not.
Bastos 2013 Brazil	RYGB D	Cross-sectional study. 64 patients, 53.4 months post-op, 89% female, avg 41 years old; pre-op BMI 49.5 kg/m^2. Interviews and questionnaires.	2 groups: > 50% EWL and < 50% EWL by 2 years post-op.	28% of patients experienced weight regain >15% of their lowest post-op weight. Working in food production (cafeteria, baker, snack bar, restaurant, grocery store) was correlated with weight regain (p = .003). Exercise (p = .075), alcohol intake (p = .572), and nutrition monitoring (p = .110) were not.
Faria 2010 Brazil	RYGB D	Intervention (non-randomized, non-blinded). 30 patients, 2–7 years post-op, 86% female, avg age 36 years old; pre-op BMI 43 kg/m^2.	3-month nutrition intervention.	Weight regain pre-intervention vs weight loss post- intervention: 8 kg vs 4.3 kg. 86% of patients had significantly lost weight and significantly reduced their BMI (p < .001) in patients with previous weight regain.
Yanos 2015 USA	RYGB D, B, E	Cross sectional. 97 patients, 8.76 years post-op, 77.3% female, 92.8% Caucasian, avg age 56.11 years; pre-op BMI: n/a. Assessed self- management behaviors, drug and alcohol use, food addiction, and physical activity.	N/A	Average of 26% weight regain from nadir weight. Significant weight regain (=20% weight regain from nadir weight) was associated with dietary adherence (p = .005-.018), physical activity modification (p = .002), nocturnal eating (p = .01), depression (p = .001), and problematic alcohol (p = .01).
Reid 2016 Canada	RYGB D	Cross sectional. 27 patients, 12.15 years post-op, 89% female, avg age 53 years old, pre-op BMI: n/a. Assessed dietary, vitamin compliance, physical activity, and follow-up.	2 groups: Weight maintainers: having lost ≥38% total body weight. Weight regainers: having lost ≤30% total body weight.	Weight loss of weight maintainers vs weight regainers: 44.4% vs 18.2%. People who regained their weight reported consuming more carbohydrates (p < .05) and alcohol (p < .05). No difference in frequency of vitamin supplementation and contact with a healthcare professional.
Himes 2015 USA	RYGB D, B	Intervention (non-randomized, non-blinded). 28 patients, avg 4 years post-op, 93% female, all Caucasian; avg age 53 years old; pre-op BMI: n/a.	6-week group CBT and DBT treatment.	Weight regain of 17 kg (37% of initial weight loss) prior to intervention. Weight loss of 1.6 kg (p ≤ .01) after the 6-week intervention. Patients reversed their pattern of weight regain (weight loss of 1.6 kg, p ≤ .01). Subjective binge eating (p ≤ .03), the number of daily snacks (3.9 to 2.7, p ≤ .01) and the number of eating episodes per day (6.7 to 5.5, p ≤ .01) significantly decreased during the intervention.
Mitchell 2016 USA	RYGB LAGB B	Cohort of 2022 patients, 3 years post-op, 78% female, avg age 47 years old; pre-op BMI n/a. Assessed dietary and lifestyle behaviors, including drug and alcohol abuse pre-op and post-op.	N/A	The factors associated with a 16% variability in weight at 3 years post RYGB are: weekly self-weighing, continuing to eat when feeling full more than once a week, and eating continuously during the day. Applying these positive habits post-op results in a 14% greater weight loss than those who don't apply them (p < .001).
McGrice 2015 USA	Sleeve gastrectomy RYGB LAGB D, B, E	Review: To review the challenges and solutions of interventions that improve long-term weight loss post bariatric surgery.	N/A	Loss of control eating, excessive energy intake, and a lack of exercise were associated with weight regain.
Lauti 2016 New Zealand	Sleeve gastrectomy S, E	Systematic review of 20–208 patients 2–9 years post-op age: n/a pre-op BMI 34.3-45.8 kg/m2. 5/21 studies addressed modifiable factors for weight regain Follow-up: 3 studies Lifestyle behavior: 2 studies	N/A	Rates of regain ranged from 5.7%at 2 years to 75.6%at 6 years. 2 studies addressing modifiable factors showed a regain of 20% EWL. Regular and frequent follow- up was associated with less weight regain, while maladaptive eating and lack of exercise was attributed to weight gain. Other non-modifiable factors were identified.
Karmali 2013 Canada	RYGB AGB VBG Sleeve gastrectomy Other D, B, S, E	Systematic review of 26–1845 patients 1–11.4 years post-op age: n/a pre-op BMI: n/a. 8/16 studies showed modifiable factors associated with weight regain.	N/A	Regain between 7.3–9% of EWL; increased BMI ~5.3 points; regain of =15% of total weight loss; regained ~22.6 lbs. Higher caloric intake, poor dietary quality, BED, grazing, depression, alcohol and substance abuse, lack of self-monitoring, little or no follow-up with team, and a lack of exercise were associated with weight regain. Other non-modifiable factors were identified.
Amundsen 2016 Norway	RYGB B, E	Case control. 40 patients, avg 5 years post-op, 82% female, avg 46 years old; pre-op BMI 44.1. Assessed dietary intake, eating behavior psychometrics, and physical activity through questionnaires.	2 groups: significant weight regain (>15%) vs normal weight regain (=15%).	Significant weight regain group: 43.7% total weight regain. Normal weight regain group: 6.8% total weight regain. Disinhibited eating (p = .015) and less exercise (p = .003) were associated with significant weight regain. Exercising for 567 min/week vs 287 min/week was associated with the group with normal weight regain.
Conceicao 2014a Portugal/USA	RYGB LAGB B	Cross sectional. 374 patients, 4 groups: pre- op, 6 months, 1 year, and 2 years post-op, 88.2% female; avg age 43.3 years old; avg pre- op BMI for all groups: 45 kg/m2. All patients assessed for maladaptive eating through self-reported questionnaires.	N/A	Higher weight regain in group with LAGB at year 1 and 2 post-op, amounting to 17.7% weight regain by year 2. Those with RYGB regained 5.5% body weight by year 2. Picking and nibbling was associated with weight regain (p < .000).
Nicolau 2015 Spain	RYGB Sleeve gastrectomy B	Cross sectional. 60 patients; 46.5 months post-op, 78.3% female, avg age 46.3 years old, pre-op BMI 48.3. Assessed dietary habits, grazing, depressive disorder and QOL via surveys and a semi-structured interview.	N/A	72% of people who grazed gained weight as opposed to people who did not graze (p < .000). Individuals who reported a grazing pattern were more prone to weight regain and achieved a lesser percentage of excess weight loss.
Pekkarinen 1994 Finland	VBG B	Cohort of 27 patients, avg 5.4 years post-op, 70.3% female, avg age 36; avg pre-op BMI: 50. Assessed dietary intake via a food record and disordered eating patterns through questionnaires further validated via a semi- structured interview.	2 groups: bingers vs non- bingers.	People who binged and people who did not binge had comparative results at 1 year post-op (55% EWL and 57% EWL, respectively), but at 2 years post-op, people who binged regained more weight than people who did not bingers (24% EWL and 50%EWL, respectively p = .04).
Conceicao 2014b Portugal	RYGB Sleeve gastrectomy LAGB B	Cross sectional. 168 patients, 3 groups: pre-op, short-term (11.4 months), and long-term (55.7 months), 88.1 % were women; and avg age 43.5 years; avg pre-op BMI 45.1 in long term group. Assessed disordered eating patterns, depression, and body image via interviews and self-reported measures.	3 groups: pre-op, <2 years, >2 years.	In long-term group (>2 years post-op) experiencing LOC, lowest BMI was 31.6 and BMI at time of study was 37.5 kg/m2. LOC eating was related with the highest BMIs, the least weight loss, most weight regain, and most psychological impairment in the long-term assessments, but not at short-term.
Rudolph 2013 Germany	RYGB LAGB VBG LGP (plication) Other S	Systematic review and meta-analysis. 13–144 patients, 0–53 months post-op, 12–100% female, 21–52.5 years old, pre-op BMI 42.6– 51.6 kg/m2. Various treatments observed: CBT, SGA (support group attendance), and physical activity.	N/A	3 studies observed weight up to 2–3 years post-op. Patients receiving behavioral management had greater weight loss than patients receiving usual care or no treatment. Papalazarou *et al.* (2010) showed that individual CBT given to VBG patients from the time of surgery and up to 3 years resulted in a maintenance of %EWL from 12 (76.4%) to 36 months (74.8%) post-op vs their controls (57.5% and 49.1%, respectively). Niazi *et al.* (2012) showed that group CBT to gastric plication patients showed 90.0 %EWL in treatment group vs 43.4%EWL in control group at 24 months post-op.
Gould 2007 USA	RYGB S	Cohort of 85 patients, 3–4 years post-op, =78% female in each group, avg age 43 years avg pre-op BMI: 50 kg/m2. Assessed for attendance to follow-up (f/u) visits.	3 groups: attended all visits for >3 years, attended all visits up to 1 year, and lost to follow-up before 1 year.	Patients who attended all scheduled follow-up appointments experienced greater long-term weight loss than those who did not (p < .05): 74% EWL in those who attended every f/u visit vs 61%EWL in those who attended f/u for first year post-op then lost to f/u vs 56% EWL in those who were lost to f/u before 1 year post-op.
Liebl 2016 USA	RYGB Sleeve gastrectomy LAGB S	Qualitative. 14 patients, 69 months post-op, 78% female, avg age 47 years, pre-op BMI: n/a, avg pre-op weight: 313 lbs. Semi-structured interviews	N/A	An average of 8% of EWL (22 lbs) was regained after surgery. To maintain weight loss, an individual must seek out and be surrounded by positive family and peer support influences. Positive support may provide the opportunity for an individual to place personal health needs as a priority, while negative influences must be identified by the individual and should be blocked out, avoided or contact may need to be eliminated.
Sarwar 2011 USA	RYGB LAGB B	Review observing potential threats posed with changes in dietary intake and eating behavior after bariatric surgery.	N/A	Clinical observations and some studies have suggested that suboptimal weight loss and other untoward outcomes (eg, nutrition, vomiting, and dumping) are often attributed to poor adherence to the postoperative diet and/or a migration to maladaptive eating behaviors. Sustained improvements in dietary intake and eating behaviors are critical to long-term success after bariatric surgery.
Silver 2006 USA	RYGB E	Cross sectional. 140 patients, 24.2 months post-op, 88.6% female, avg age 45 years pre-op BMI 49.8 kg/m2. Assessed behaviors, dietary behaviors, and physical activity via questionnaires.	N/A	A higher BMI at 2 years post-op was associated with less exercise (P=0.006). 82.9% continued to be physically active in an effort to lose or maintain weight, with 62.9% engaging in physical activity at least 3x/wk, with an average duration of 54.7 minutes.
Livhits 2010 USA	RYGB LAGB VBG DS E	Systematic review. 30–1585 patients, 18.9 months post-op, 'mostly' female, avg age 44 years, avg pre-op BMI 47.5.	N/A	6/13 studies observed exercise ≤16 months post- op. Meta-analysis suggests exercise results in a 4.2% greater degree of weight loss at 12 months, and greater weight loss persists out to 24 months. While Bond *et al.* (2004) report that patients who were active lost more weight than those who were sedentary 24 month after surgery, Larsen *et al.* (2006) found no evidence of a beneficial association at 24 months post-op.
Hsu 1998 USA	RYGB VBG B	Review.	N/A	Binge eating behavior and low metabolic energy expenditure, are associated with weight regain.

D = dietary, B = behavioral, avg = average, BMI = body mass index, BED = binge eating disorder, RYGB = Roux-en-Y gastric bypass, EWL = excess weight loss, E = exercise, LAGB = laparoscopic adjustable gastric band, S = support, VBG = vertical banded gastroplasty, QOL = quality of life, LGP = laparoscopic gastric plication, DS = duodenal switch

**Figure 2.**
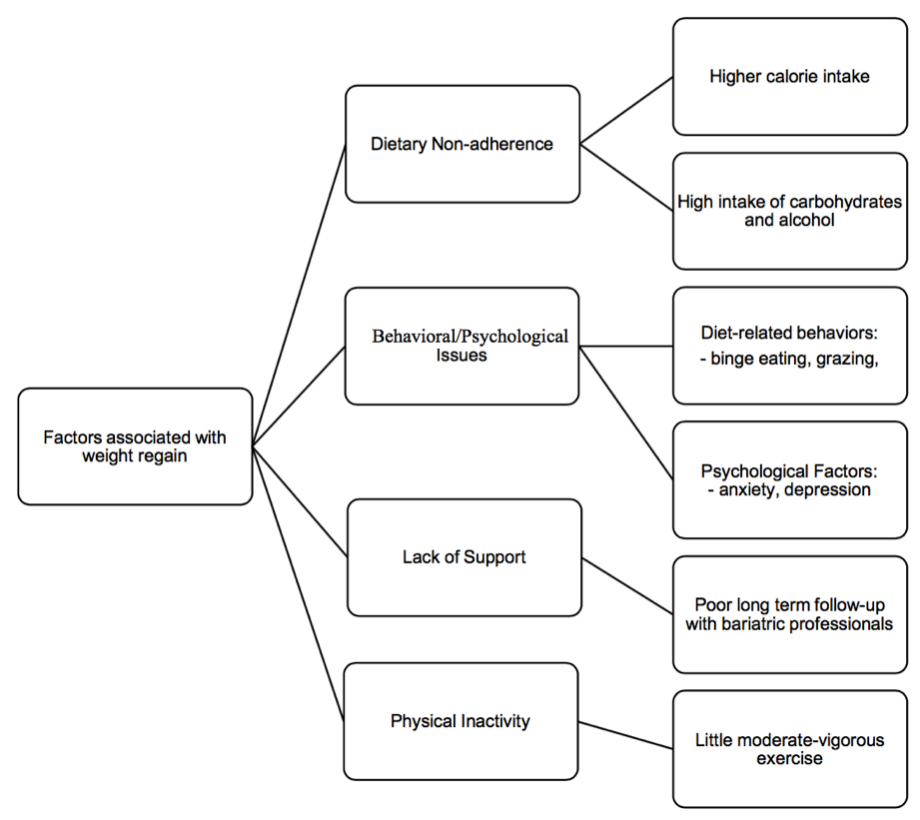
Conceptual framework. This conceptual framework depicts the factors associated with weight regain including dietary non-adherence, behavioral/psychological issues, lack of support, and physical inactivity, as well as the subgroups specific to each factor.

Three of the authors (LK, AH, MJ) independently reviewed the studies and organized the findings into themes, based on the types of modifiable behavior examined (see Figure 2). Weight regain after bariatric surgery was found to be multifactorial, resulting from an interplay of different modifiable behaviors—including poor dietary adherence, behavior/psychological issues, lack of support and physical inactivity. The following sections provide a critical review of each of these themes in more detail. While weight regain was defined and expressed differently across each study, behavioral differences that were associated with weight regain were compiled into themes.

### Poor dietary adherence

 Of the 22 studies included in our thematic analysis, eight (36%) ^8, 12– 18^ suggested that diet was significantly associated with weight regain (p = 0.001-0.05) after bariatric surgery. Poor adherence to dietary guidelines - represented by higher carbohydrate intake ^16^, higher alcohol intake ^8, 15, 16^ and lower dietary quality ^8^ - were key contributors.

Two studies reported that a higher energy intake (2000 vs. 1500 Kcal/day) was associated with a weight regain of 11 kg from nadir weight as of two years after surgery ^8, 12^ Similarly, Himes *et al.* ^17^ reported a 10% weight loss of the total weight regained in as little as six weeks by reducing the frequency of eating episodes from 6.7 to 5.5 episodes, and 86% of participants in Faria *et al.* ^14^ lost 54% of their weight regain in three months following a 1400 Kcal/day prescription. This suggests that a higher frequency of eating episodes and higher energy intake over time may have contributed to weight regain prior to the interventions.

In terms of dietary quality and alcohol intake, Reid *et al.* ^16^ observed a 26% difference in weight outcomes (p ≤ 0.01), at 12 years post-operation, between people who maintained their weight and people who regained some weight. In this study, people who regained some weight consumed more carbohydrates than people who maintained their weight (222 vs. 162 g/day, p < 0.05); however, there was no difference in the percentage of energy intake from carbohydrates in both groups (43% vs. 42%, respectively) ^16^ While people who regained some weight consumed more alcohol than people who maintained their weight (1.32 vs. 0.19 standard drinks/day; p < 0.05) in Reid *et al.*’s study ^16^ reported consumption was still within the suggested limits for the general population. Other studies, however, have determined that alcohol misuse or abuse was associated with weight regain ^8, 15^. A higher median fat intake was observed in people who regained some weight (88.6 vs. 64.3 g/d, p < 0.05), although the percentage of energy intake from fats was similar in both groups (41.7% vs. 37.4%, respectively) ^12^. Similarly, Karmali *et al*. refer to studies that reported a higher energy intake ^19^ and poor dietary quality ^3^, including higher sugar, sweets and fatty foods, were attributable to weight regain as of two years after RYGB, VBG, and adjustable gastric band (AGB).

Finally, Bastos *et al*. examined the influence of having a food-related occupation on weight regain. They determined that working in food production - whether as a baker or working in a cafeteria, snack bar, restaurant, or grocery store - was correlated with significant weight regain (p = 0.003) ^13^.

Of the eight studies that observed diet as a factor associated with weight regain after bariatric surgery, seven of them observed calorie intake as a contributing factor. A higher calorie intake - whether from carbohydrates, alcohol, low nutritive value sweets, fatty foods, or as a result of a higher frequency of eating episodes - are associated with weight regain ^8, 12– 18^. Only one study observed the association of working in the food industry with weight regain. Contrary to other studies, however, they did not observe alcohol intake as a contributing factor.

### Behavioral/psychological issues

Thirteen studies (59%) identified post-operative diet-related behaviors, or eating habits, and psychological factors were associated with long-term post-operative weight regain.

***Maladaptive eating behaviors.*** Of the 12 studies that examined diet-related behaviors, nine studies (75%) found a significant association with post-operative weight regain ^8, 18, 20– 27^. Variations of these habits, including binge eating, disinhibited eating, picking and nibbling or grazing, and loss of control eating behaviors, have contributed to a weight regain ranging from 10 kg to 17 kg ^8, 17^, nearly 11% gain from nadir weight at two years after surgery ^22^. At five years post-operative, weight regain was found to be as high as 44% from nadir weight, as a result of disinhibited eating, or the tendency to overeat, and unsuitable eating behaviors ^21^. While most studies have demonstrated the association of maladaptive dietary behaviors with weight regain ^8, 18, 20– 27^, Himes *et al.* ^17^ demonstrated that these behaviors improved with interventions; for example, a group behavioral cognitive behavioral therapy (CBT) intervention led to a 1.6 kg weight loss over six weeks in those who had been on a weight regain trend post bariatric surgery. Meanwhile, Mitchell *et al.* ^20^ determined that grazing and binging habits, in addition to a lack of self-monitoring, accounts for 16% variability in weight outcomes. Pekkarinen *et al.* ^24^ showed that although people who binged and people who did not binge had similar outcomes at one year post-VBG (55% vs. 57%, respectively), people who binged regained significantly more weight than people who did not binge two years after their operation (24% vs. 50%, respectively, p = 0.04). Finally, Hsu *et al.* ^27^ showed that those who suffered from binge-eating disorder (BED) prior to surgery continued to struggle with this after bariatric surgery. This finding was further confirmed by Colles *et al.,* ^28^ who found that grazing habits increased post bariatric surgery (26% pre-operation to 38% by one year post-operative), while Nicolau *et al.* determined that 72% of people who grazed gained weight vs. people who did not graze (p < 0.0001) ^23^. However, Alvarez *et al*. did not find BED to be associated with post-operative weight regain.

Of all 22 studies reviewed, only one had identified nocturnal eating as an important determinant of weight regain (p = 0.01) ^15^.

***Psychological factors.*** Three articles determined that depression ^8, 15^, anxiety ^12^, and alcohol and/or substance abuse ^8, 12, 15^ were associated with post-operative weight regain.

### Lack of support

Five articles (23%) suggested poor post-operative support was associated with long-term weight regain ^8, 29– 32^. Four of these studies examined the impact of follow-ups with a bariatric team; they determined that little to no post-operative follow-up can lead to poor long-term outcomes ^8, 29– 31^. Those who maintained regular follow-up, for up to three years post-operation, had better long-term weight outcomes (74% excess weight loss, EWL) ^30, 31^ than those who were lost to follow-up within the first year of their surgery (56% EWL, p < 0.05) ^30^. Among the 75% of patients who no longer received follow-up as of three years post-operation, they were observed to regain up to 14% more weight; in comparison, only 25% of patients who received an annual follow-up up to five to six years post-operation were found to experience weight regain ^29^. In another study, regular follow-up represented 47% difference in %EWL at two years post-operation ^30^.

Post-operative support from health care professionals was found to be an important component in long-term success. Karmali *et al.* ^8^ cited that, among those who failed surgery, 60% had never seen a dietitian and 80% had never seen a psychologist. Comparatively, Gould *et al.* ^31^ reported greater long-term outcomes in patients who attended all post-operative follow-up visits with a multidisciplinary team. A post-operative bariatric surgery patient who received individual or group CBT sessions had a higher %EWL (90% vs. 43% EWL) at two years post-surgery and better weight loss outcomes than the controls who did not receive support ^30^. Yet, despite the known benefits of post-operative support, there was little evidence in the literature in support group attendance and its influence on weight regain or weight maintenance. Liebl’s qualitative study ^32^ described the experiences of post-operative bariatric patients who were successful at maintaining weight loss (average of 8% of EWL regained) at an average of 69 months post-surgery. The patients surveyed in the study reported that support from their family, peers and professionals from their bariatric surgery clinic had been necessary to achieve positive outcomes.

### Physical inactivity

Seven studies (32%) addressed the relationship between physical activity and weight regain. Questionnaires, which primarily captured moderate to vigorous intensity activity, were used to determine activity levels in three of the studies ^15, 18, 21^. Four systematic reviews reinforced that lower physical activity levels were associated with poorer weight loss outcomes and higher weight regain, despite undergoing bariatric surgery ^8, 18, 29, 33, 34^. Furthermore, Amundsen *et al.* ^21^ observed that people who significantly regained weight (total weight regain ≥ 15% from nadir weight), were less active than people who regained a normal amount of weight (p=0.06 *)*. People who maintained their weight spent remarkably more time being moderately active (567 min per week) compared to people who regained weight (287 min per week). 

## Discussion

In this scoping review, we have presented a summary of the existing literature on modifiable factors associated with weight regain after bariatric surgery - namely, poor dietary adherence, behavioral and psychological issues, lack of support, and physical inactivity - and highlight their potential relevance to patients and practicing healthcare professionals in this field.

### Poor dietary adherence

Studies related to dietary adherence suggested that poor observance of the dietary guidelines -represented by higher carbohydrate intake, higher alcohol intake and lower dietary quality - were key contributors to weight regain in the long-term recovery from bariatric surgery.

Changes in dietary adherence over the course of the post-operative phase may be associated with weight regain in patients after bariatric surgery ^8, 12, 16, 18^. Higher carbohydrate consumption appears to be the most evident dietary cause associated with weight regain ^16^. Although the source of carbohydrates was not clearly defined in all studies, some have demonstrated that an increased consumption of liquid calories and sugar intake from non-nutritive sources were attributable to weight regain ^26^. Thus, increases in patient’s non-nutritive, free- and added-sugar intake potentially explain some of the weight regain following bariatric surgery.

Alcohol is a source of empty liquid calories which contributes significantly to one’s caloric intake, and some studies have found a positive association between weight regain and alcohol abuse or misuse ^8, 15, 16^. This is particularly concerning because alcohol abuse has been shown to be higher among people who have had bariatric surgery, in comparison to the general population ^35^. While there does not appear to be a consensus on post-operative weight regain and alcohol intake, one of the studies reported an association between intake levels at or beneath the suggested alcohol intake limits ^16, 36^. The discrepancies seen in the literature may be due to the nature of the data collection. Alcohol intake is known to be underreported by up to 50% when self-reported ^37^. This is especially true among middle-aged women, which coincides with the demographic of the bariatric population ^37^. Patients need to be educated on the effects of alcohol consumption after malabsorptive bariatric procedures due to changes in alcohol metabolism, particularly after a gastric bypass. Studies have demonstrated an acceleration in alcohol absorption after a gastric bypass such that it takes a shorter time to reach a maximum concentration. In addition, there is a higher maximum alcohol concentration achieved, as well as it taking longer to fully metabolize and eliminate alcohol ^38^. As such, despite the inconclusive results, results from this review suggest alcohol intake should be more closely assessed and monitored in the people who have had bariatric surgery, even if their consumption is within the suggested limits according to the Centers for Disease Control and Prevention ^36^.

Given the gastric restriction of bariatric surgery, increased calorie intake occurs in the form of more frequent eating episodes and/or in the consumption of more calorie-dense foods or liquid calories. This demonstrates that dietary patterns after bariatric surgery do not remain consistent; rather, there is a gradual onset of undesirable dietary habits that develops, and some patients may not be cognizant of the effects this will have on their future outcome. Therefore, bariatric surgery alone is not protective in the long term; patients will likely require the ongoing support and monitoring of dietitians and their bariatric team to find solutions and alternatives to the challenges to maintain adherence to dietary recommendations. 

### Behavioral/psychological issues

Behavioral and psychological factors may impede one’s ability to comply with post-operative lifestyle recommendations. While restrictive and malabsorptive procedures may limit the amount of food consumed in a given sitting, it does not generally limit the ability to eat significant volumes over the course of a day. Grazing and binging were the most commonly identified eating behaviors associated with weight regain. All, except for one study, clearly observed this relationship. Although maladaptive eating habits do not negatively affect one’s weight outcomes at one year post-operation, people who continue to binge have a higher risk of regaining weight by the second year following surgery ^24^. Furthermore, Himes *et al.* ^17^ suggests that therapy aimed at reducing binge eating behaviors can lower the number of daily eating episodes and encourage weight loss following weight regain. Therefore, targeted therapy towards maladaptive eating behaviors provided early on in the patient’s recovery process may help to prevent occurrence of weight regain. 

Given that maladaptive eating behaviors that exist prior to the surgery appear to remain an issue in the post-operative phase ^27^, dietary behaviors are an important target point for discussion even before the weight loss surgery is performed. Health professionals should educate patients on the evolution of maladaptive eating behaviors after surgery to encourage therapy and treatment prior to the operation and on an ongoing basis. Professionals should continue to monitor and probe for various types of maladaptive eating behaviors long-term after surgery.

Most of the articles that studied behaviors looked at maladaptive eating behaviors also found lower levels of physical activity as contributing factor to weight regain. This suggests that there may be a relationship―and, potentially, interactive or cumulative impacts―between eating behaviors, dietary intake and physical activity.

### Lack of support

The American Society for Metabolic and Bariatric Surgery (ASMBS) has produced guidelines on post-operative follow-up care with a bariatric team, which emphasizes the importance of follow-up care on a patient’s progress and outcomes. Follow-up care helps to track the patient’s progress, ensure their adherence to recommendations made by their health care team and offers solutions for managing weight and lifestyle. With 50% of people who have had bariatric surgery regaining weight within two years of their surgery, it is not surprising that 60% of these patients had not received regular nutrition follow-up and 80% did not receive psychological follow-up ^39^; in contrast, patients who attended all of their post-operative follow-up were more successful in the long-term ^31^. Follow-up maintained for up to three years post-operation resulted in 18% higher EWL compared to a patient who was lost to follow-up within the first year of surgery ^31^, which may infer a positive correlation between longer follow-up periods and improved health outcomes. The impact of support has been well studied post bariatric surgery, as evidenced by several systematic reviews on the subject. Unfortunately, studies included in this scoping review were only able to highlight the benefit of post-operative support with a bariatric team; it was not clear from the literature if there is a single member of the bariatric team that is pivotal in providing the follow-up, even though several studies observed follow-up with either a dietitian or a psychologist.

From the patient’s perspective, positive family, peer, and professional bariatric support were identified as being vital in achieving long-term post-operative success. This offers important insight into the patients’ perspectives and the three prongs of support considered necessary to optimize their long-term outcomes. Based on these findings, a patient’s health care provider and social network - which may include family members and friends - should encourage the patient to present to follow-up appointments and, ideally, accompanied by their family, partners or friends. Given that patients usually forget the information they have been taught prior to surgery, which further worsens at one year post-operation ^40^, inviting the patients’ support systems to follow-up appointments may encourage them to maintain regular contact with their bariatric team and also serve as an aid to remind patients of the positive interventions and behaviors discussed during consultations.

### Physical inactivity

Bariatric surgery could greatly improve mobility and reduces the burden of osteoarthritis. As previously supported in the literature, participants with obesity tend to over-report their physical activity levels ^41^. While 89% of patients self-reported to be regularly active at two years post-operation ^33^, other studies observed that only about half of patients were engaged in moderate to vigorous activity for more than one session per week ^42^, which is lower than the activity of adults in the general population (48% vs 53%, respectively) ^43^. Furthermore, only 11% ^16^ of people who have had bariatric surgery, as compared to 35% ^43^ of the general population, actually achieve 10,000 steps per day when measured using accelerometers.

Although there were several systematic reviews on the impact of physical activity on post-operative bariatric surgery outcomes, there is little information available on the subject of physical activity and weight regain; most articles cite that ‘low energy expenditure’ was associated with weight regain. It has also been suggested that current physical activity guidelines may be too low to prevent long-term weight regain after bariatric surgery ^21^. However, it is observed that despite having similar moderate to vigorous physical activity habits to the general population, people who have had bariatric surgery are less active on a daily basis.

### Limitations in the literature

There were several identified gaps in the literature, which could limit the generalizability of the conclusions presented here. Firstly, there is not a consistent definition and measure for “weight regain”, or a clear indication of what is considered to be normal weight regain. This makes it difficult to compare the effect size across studies and modifiable factors in relation to weight regain. The ASMBS has suggested standardized outcomes reporting; however, the association has not offered guidelines on reporting magnitude of weight regain. In addition, although participants were stratified into groups, the cut-off points used in some studies to describe percentage of weight regain may not be sensitive enough (i.e. >50% EWL vs ≤50% EWL) to observe clinically significant changes. As a result, there may have been poor differentiation between outliers and participants who narrowly fell within the cut-off range ^12^.

Secondly, the surveys and questionnaires used were unique to each study, resulting in inconsistent metrics that are often not directly comparable. This is important since different surveys for assessing lifestyle habits may lead to varying results and conclusions. The use of different tools and questionnaires that have not been validated for the bariatric surgery population may also explain the discrepancies observed for the expected similar associations.

Thirdly, the majority of the studies relied on self-reported measures of modifiable behaviors. Dietary misreporting in the population affected by obesity is particularly concerning and has been well-documented in a previous study, such that people who under-reported their energy intake were more likely to be inflicted with obesity ^44^. Similar results have been observed for self-reported physical activity, which is generally over-reported. All but one study included in this scoping review relied on questionnaires and self-reported data; hence, making it difficult to reach a robust conclusion on physical activity levels post-surgery. Therefore, self-reported data inevitably limits the external validity of the findings.

Another source of variability to consider is that this scoping review included all types of bariatric surgery, even though the VBG is no longer performed and the AGB is being phased out as a routine procedure. However, even though each procedure is different in the weight loss achieved and outcome experiences (with regards to tolerance of certain foods), the modifiable behaviors would be similar across surgery types.

Lastly, most of the studies included in this scoping review were observational. However, even among the interventional studies, the sample sizes were very small (n<30), making it difficult to determine the true cause and effect.

## Conclusion

Although effective, weight regain can still occur after bariatric surgery. Findings of this article support the notion that people who have had bariatric surgery need to be informed of the modifiable factors associated with weight regain in an effort to encourage long-term weight loss maintenance. Regular, routine and long-term follow-up with the bariatric team is essential to the long-term weight regain prevention. Follow-up support may act as a pivot to addressing poor dietary adherence, behavioral issues and physical inactivity that impact long-term weight outcomes in a timely manner.

Future research should identify a common definition and measurement for weight regain post-bariatric surgery and agree upon accepted surveys and questionnaires validated in the bariatric population. Future research should also identify the specific foods, eating frequency and type of physical activity that may be the most relevant to people who have had bariatric surgery to provide healthcare professionals with a better understanding of the types of foods to suggest to limit and the types of activities to reinforce. The literature can benefit from more randomized clinical trials targeting dietary protocols and patient support that include better controls. Finally, rigorous subgroup analyses to enable comparison of outcomes and relevant interventions among patients undergoing different procedures, as well as among those who suffer from severe obesity (BMI 35–49) and super-severe obesity (BMI ≥50), will be important for personalized care planning in this patient population.

## Data availability

### Underlying data

All data underlying the results are available as part of the article and no additional source data are required.

### Extended data

Open Science Framework: Modifiable Factors Associated with Weight Regain After Bariatric Surgery: A Scoping Review. https://doi.org/10.17605/OSF.IO/9Q78A ^11^.

This project contains the following extended data:

- Boolean Search Terms.docx

Data are available under the terms of the Creative Commons Zero "No rights reserved" data waiver (CC0 1.0 Public domain dedication).
