# Supplementary material for: Modifiable factors associated with weight regain after bariatric surgery: a scoping review
Source: F1000Res. 2020 Sep 3;8:615. Originally published 2019 May 3. [Version 2] doi: 10.12688/f1000research.18787.2 (PMC7492783; doi:10.12688/f1000research.18787.2)
Supplement: Peer review history

# Peer review: Reviewer response for version 2

**DOI:** 10.5256/f1000research.29138.r70741

**Published:** 2020-09-15

**Authors:** Boris Zevin

**Affiliations:** 1 Department of Surgery, Queen’s University, Kingston, ON, Canada

Dear authors,

 Thank you very much for your comprehensive responses to my prior comments and suggestions. I have no further recommendations. 

 Boris Zevin MD, PhD

Is the work clearly and accurately presented and does it cite the current literature?

Yes

If applicable, is the statistical analysis and its interpretation appropriate?

Not applicable

Are all the source data underlying the results available to ensure full reproducibility?

Partly

Is the study design appropriate and is the work technically sound?

Yes

Are the conclusions drawn adequately supported by the results?

Partly

Are sufficient details of methods and analysis provided to allow replication by others?

Yes

Reviewer Expertise:

Bariatric and Metabolic Surgery

I confirm that I have read this submission and believe that I have an appropriate level of expertise to confirm that it is of an acceptable scientific standard.

# Peer review: Reviewer response for version 1

**DOI:** 10.5256/f1000research.20582.r52485

**Published:** 2019-09-30

**Authors:** Boris Zevin

**Affiliations:** 1 Department of Surgery, Queen’s University, Kingston, ON, Canada

Thank you very much for the opportunity to review this manuscript.  The authors conducted a scoping review of the literature from 1990 to 2017 identify the modifiable factors associated with weight regain following bariatric surgery.  I commend the authors on exploring this important topic as the issue of weight regain after bariatric surgery is gaining increased importance with over 215,000 primary bariatric procedures performed per year in USA ^1^.  

 To help situate this manuscript in the current literature, I would suggest for the authors to define two distinct patient populations following bariatric surgery: (1) individuals who were initially successful in losing weight and have regained some or all of weight previously lost, and (2) individuals who have failed to lose the appropriate percentage of weight post-op.  These two patient populations are different and respond differently to additional weight loss interventions.  Defining these would help the readers understand which patients this review is focused on (i.e. # 1).  

 I would encourage the authors to better define weight regain in this manuscript.  At present, it is unclear what cut-off is being used to define weight regain (i.e. gaining 5% of maximum weight lost vs regaining all of the weight previously lost).  A recent study in JAMA suggested reporting weight regain as percentage of maximum weight lost as this parameter appeared to perform better for association with most clinical outcomes than the alternatives examined [2].

 In addition, I would encourage the authors to write a paragraph in the introduction section of the manuscript discussing obesity as a chronic disease, which, as other chronic diseases, will require multiple interventions over the person's lifetime to treat.   The Obesity Medicine Association defined obesity as "a chronic, relapsing, multifactorial, neurobehavioral disease, wherein an increase in body fat promotes adipose tissue dysfunction and abnormal fat mass physical forces, resulting in adverse metabolic, biomechanical, and psychosocial health consequences."  Framing your discussion around this contemporary definition of obesity would help the readers understand why lifestyle, pharmacotherapy and surgery are all important treatments for patients with obesity. 

 Lastly, I would like to make a couple of suggestions for minor revisions to the manuscript: 

1. Introduction: The authors note that "Bariatric surgery has been established as a promising treatment option for severe obesity "; however, I would argue that bariatric surgery is a proven treatment for severe obesity with over 20 years of outcomes data.  I suggest revising the wording.
2. Introduction: The authors report that "Studies have estimated an average of 56% of patients regain weight within two years of their surgery, and about one in four fail to achieve the average expected weight loss and begin to regain weight from their lowest post-operative weight, following the first post-operative year."  This statement needs to be clarified in the context of my prior comments about the definition of weight regain. In the current form, it makes it seem that most of the patients regain weight after surgery, suggesting that surgery is not effective.  This is false and can contribute to the biases among the health care providers that surgery is not effective.   In a recent study from Kothari and colleagues, reported on 10 year follow-up post bariatric surgery on > 70% of patients ^3^.  They reported a 56% EWL at 10 years post gastric bypass - confirming durability of the results after this operation.
3. In the discussion section, please consider discussing why patients should be educated about abstinence from alcohol after malabsorptive bariatric operations (in addition to possible weight regain) and changes in alcohol metabolism post gastric bypass.
4. Discussion: Given the finding of frequency of follow-up being associated with weight loss outcomes, please consider discussing how the health care system and health care providers can address the need for ongoing follow-up of bariatric surgery patients in a resource constrained environment of a publicly funded Canadian health care system?  Why should be following these patients?  How long and how often?

Is the work clearly and accurately presented and does it cite the current literature?

Yes

If applicable, is the statistical analysis and its interpretation appropriate?

Not applicable

Are all the source data underlying the results available to ensure full reproducibility?

Partly

Is the study design appropriate and is the work technically sound?

Yes

Are the conclusions drawn adequately supported by the results?

Partly

Are sufficient details of methods and analysis provided to allow replication by others?

Yes

Reviewer Expertise:

Bariatric and Metabolic Surgery

I confirm that I have read this submission and believe that I have an appropriate level of expertise to confirm that it is of an acceptable scientific standard, however I have significant reservations, as outlined above.

## References

[1] : Comparison of the Performance of Common Measures of Weight Regain After Bariatric Surgery for Association With Clinical Outcomes. *JAMA*.2018;320(15) : 10.1001/jama.2018.14433 10.1001/jama.2018.14433 PMC623379530326125

[2] : American Society for Metabolic and Bariatric Surgery estimation of metabolic and bariatric procedures performed in the United States in 2016. *Surg Obes Relat Dis*.14(3) : 10.1016/j.soard.2017.12.013 259-263 10.1016/j.soard.2017.12.013 29370995

[3] : Long-term (>10-year) outcomes after laparoscopic Roux-en-Y gastric bypass. *Surg Obes Relat Dis*.2017;13(6) : 10.1016/j.soard.2016.12.011 972-978 10.1016/j.soard.2016.12.011 28223086

# Response

**Published:** 2020-08-25

**Authors:** Lisa Kaouk

**Affiliations:** McGill University Health Centre, Canada

Dear F1000 Editors and Dr. Zevin,

Thank you for facilitating the peer-review process. We have uploaded a revised copy of the manuscript and have addressed all of Dr. Zevin’s comments to further improve the clarity and quality of our paper. Please find our responses to each comment below.

1.   “To help situate this manuscript in the current literature, I would suggest for the authors to define two distinct patient populations following bariatric surgery: (1) individuals who were initially successful in losing weight and have regained some or all of weight previously lost, and (2) individuals who have failed to lose the appropriate percentage of weight post-op.  These two patient populations are different and respond differently to additional weight loss interventions.  Defining these would help the readers understand which patients this review is focused on (i.e. # 1).” 

We have added the following in the introduction:

There are two distinct patient populations observed with people who had bariatric surgery with suboptimal or poor outcomes. There are those who do not lose the expected or anticipated average percentage of weight following surgery, while there are others who lose a successful amount of weight but who regain some, or most, of the weight they had initially lost via bariatric surgery. This scoping review observes the latter group of people.

2.  “I would encourage the authors to better define weight regain in this manuscript.  At present, it is unclear what cut-off is being used to define weight regain (i.e. gaining 5% of maximum weight lost vs regaining all of the weight previously lost).  A recent study in JAMA suggested reporting weight regain as percentage of maximum weight lost as this parameter appeared to perform better for association with most clinical outcomes than the alternatives examined [2].” 

We have added the following in the Inclusion and exclusion criteria of the Methods section.”

Given that the research community has not yet reached consensus on how weight regain is defined or compared, all studies addressing weight regain either as a percentage of regain or in weight gained were included regardless of the extent of regain.

3.  “In addition, I would encourage the authors to write a paragraph in the introduction section of the manuscript discussing obesity as a chronic disease, which, as other chronic diseases, will require multiple interventions over the person's lifetime to treat.   The Obesity Medicine Association defined obesity as "a chronic, relapsing, multifactorial, neurobehavioral disease, wherein an increase in body fat promotes adipose tissue dysfunction and abnormal fat mass physical forces, resulting in adverse metabolic, biomechanical, and psychosocial health consequences."  Framing your discussion around this contemporary definition of obesity would help the readers understand why lifestyle, pharmacotherapy and surgery are all important treatments for patients with obesity.” 

We have added the following to the introduction:

Before discussing bariatric surgery, it would be important to first address obesity as a complex chronic disease which requires several interventions over the course one a person’s lifetime in an effort to treat it. The Obesity Medicine Association (OMA) defines obesity as “a chronic, relapsing, multifactorial, neurobehavioral disease, wherein an increase in body fat promotes adipose tissue dysfunction and abnormal fat mass physical forces, resulting in adverse metabolic, biomechanical, and psychosocial health consequences”. As such, the treatment of obesity requires a multifaceted approach customized to each individual’s needs including treatment options such as lifestyle modifications, pharmacotherapy, and surgery, are valuable treatments. 

4.   “Introduction: The authors note that "Bariatric surgery has been established as a promising treatment option for severe obesity "; however, I would argue that bariatric surgery is a proven treatment for severe obesity with over 20 years of outcomes data.  I suggest revising the wording.” 

The change has been made.

5.  “Introduction: The authors report that "Studies have estimated an average of 56% of patients regain weight within two years of their surgery, and about one in four fail to achieve the average expected weight loss and begin to regain weight from their lowest post-operative weight, following the first post-operative year."  This statement needs to be clarified in the context of my prior comments about the definition of weight regain. In the current form, it makes it seem that most of the patients regain weight after surgery, suggesting that surgery is not effective.  This is false and can contribute to the biases among the health care providers that surgery is not effective.   In a recent study from Kothari and colleagues, reported on 10 year follow-up post bariatric surgery on > 70% of patients ^3^.  They reported a 56% EWL at 10 years post gastric bypass - confirming durability of the results after this operation.” 

Upon reading our reference #3, it appears that we misread the statement, and in fact, what Dr. Zevin had suggested is correct. As such, we maintained the reference originally used in the scoping review and made the numerical correction to properly cite the reference originally used to indicate that 56% of people regain some weight after a period of 10 years.

 6.  “In the discussion section, please consider discussing why patients should be educated about abstinence from alcohol after malabsorptive bariatric operations (in addition to possible weight regain) and changes in alcohol metabolism post gastric bypass.” 

We added the following to the discussion section:

Patients need to be educated on the effects of alcohol consumption after malabsorptive bariatric procedures due to changes in alcohol metabolism, particularly after a gastric bypass. Studies have demonstrated an acceleration in alcohol absorption after a gastric bypass such that it takes a shorter time to reach a maximum concentration. In addition, there is a higher maximum alcohol concentration achieved, as well as it taking longer to fully metabolize and eliminate alcohol. ^43^

However, I did not address that patients need to be educated about abstinence of alcohol as ASMBS’ position statement on alcohol use prior to and after surgery does not recommend abstinence post-operatively. Rather they advise patient education to inform patients of the risks of alcohol misuse post-op. While several bariatric programs across North American advise abstinence of alcohol after surgery, given that this is not a recommendation of the ASMBS, we felt it best to follow their guidelines at this time. 

https://asmbs.org/app/uploads/2016/03/SOARD-AlcoholStatement_Published_Feb2016.pdf

7.  “Discussion: Given the finding of frequency of follow-up being associated with weight loss outcomes, please consider discussing how the health care system and health care providers can address the need for ongoing follow-up of bariatric surgery patients in a resource constrained environment of a publicly funded Canadian health care system?  Why should be following these patients?  How long and how often?” 

We agree that this is an important discussion to have. However, given that our scoping review provides context not only in Canada but internationally, we feel it best not to acknowledge the public Canadian healthcare system, which differs from the US, Brazil, Europe, Australia, and Middle Eastern contexts. In addition, given that the provincial health ministries in Canada regulate their own healthcare systems, there are differences in practices, staffing, and follow-up availability across provinces. These differences are even seen within bariatric centers in the same provinces and even in the same cities. Therefore, we feel that keeping an open context to our scoping review may be of more interest to readers on an international level and are relevant across the globe, such that the references used in this scoping review were influenced by international studies.

# Peer review: Reviewer response for version 1

**DOI:** 10.5256/f1000research.20582.r49560

**Published:** 2019-06-24

**Authors:** Robert Kushner

**Affiliations:** 1 Northwestern University Feinberg School of Medicine, Chicago, IL, USA

The authors performed a systematic review using multiple databases for modifiable factors associated with weight regain following all bariatric surgery procedures. The time frame of the search, search terms and selection process for the studies appears appropriate. Twenty-two studies were included in the analysis. All articles were from 2007 to 2016 except for one (a 1994 report with VBG – a procedure that is no longer performed). The authors categorized the articles into 4 general themes. The limitations cited are appropriate.

 Table 1 displays all studies with key information included (year, type of surgery and modifiable behaviour, study design and population, intervention and group comparisons, and results). Figure 1 is a PRISMA flowchart, figure 2 is a conceptual framework. All appropriate for a review article.

**Comments:**

1. The topic reviewed is important and useful for clinicians and clinical researchers. It also provides the most up-to-date studies. However, there is a noticeable dearth of studies that focus on prospective interventions. Past reviews have included modifiable factors and interventions. ^1^^, ^^2^^, ^^3^ This is an important point since few, if any, interventions have been found to be effective in preventing or treating weight regain. So, simply laying out modifiable factors without including clinical research that has attempted to intervene is unsatisfying.
  This becomes somewhat problematic for the authors. There are several instances where they include interventions, such as citing the Hines et al (ref 17) or McGrice et al (ref 18), but few if any other interventions. It is also problematic when recommendations are given, e.g, “Regular, routine and long-term follow-up with the bariatric team is essential to the long-term weight regain prevention.” Although this is sound advice, there is a large body of literature that has attempted to understand which interventions should be provided, when to intervene, for how long and by whom. As noted earlier, interventions have been largely disappointing.
2. In the introduction, severe obesity is defined as a BMI ≥40 kg/m2, not ≥35 as stated. It is more clinically useful to express the impact of weight regain as ‘percent of weight regained from nadir’ versus ‘an average of x% of their total body weight…’ The statement that 85% of patients experience weight regain at 5 years post-surgery sounds dramatic, but has little meaning if it is not defined by more criteria. Essentially all people experience some weight regain regardless of the treatment modality.
3. It is also noteworthy that the 4 behavioural modifiable domains identified by the authors are the same that are associated with any intervention for obesity. These are not unique to the bariatric surgery patient. This may be mentioned in the paper.

Is the work clearly and accurately presented and does it cite the current literature?

Yes

If applicable, is the statistical analysis and its interpretation appropriate?

Yes

Are all the source data underlying the results available to ensure full reproducibility?

Yes

Is the study design appropriate and is the work technically sound?

Yes

Are the conclusions drawn adequately supported by the results?

Yes

Are sufficient details of methods and analysis provided to allow replication by others?

Yes

Reviewer Expertise:

NA

I confirm that I have read this submission and believe that I have an appropriate level of expertise to confirm that it is of an acceptable scientific standard.

## References

[1] : Post-operative behavioural management in bariatric surgery: a systematic review and meta-analysis of randomized controlled trials. *Obes Rev*.2013;14(4) : 10.1111/obr.12013 292-302 10.1111/obr.12013 23294936

[2] : Do postoperative psychotherapeutic interventions and support groups influence weight loss following bariatric surgery? A systematic review and meta-analysis of randomized and nonrandomized trials. *Obes Surg*.2012;22(11) : 10.1007/s11695-012-0739-4 1790-7 10.1007/s11695-012-0739-4 22930073

[3] : Prevention of Weight Regain Following Bariatric Surgery. *Curr Obes Rep*.2015;4(2) : 10.1007/s13679-015-0146-y 198-206 10.1007/s13679-015-0146-y 26627215
